# Indigenous cellulolytic aerobic and facultative anaerobic bacterial community enhanced the composting of rice straw and chicken manure with biochar addition

**DOI:** 10.1038/s41598-022-09789-3

**Published:** 2022-04-08

**Authors:** Mohd Huzairi Mohd Zainudin, Jamuna Thurai Singam, Awis Qurni Sazili, Yoshihito Shirai, Mohd Ali Hassan

**Affiliations:** 1 Laboratory of Sustainable Animal Production and Biodiversity, Institute of Tropical Agriculture and Food Security, Universiti Putra Malaysia (UPM), 43400 Serdang, Selangor Malaysia; 2 Department of Animal Science, Faculty of Agriculture, Universiti Putra Malaysia (UPM), 43400 Serdang, Selangor Malaysia; 3 Department of Biological Function and Engineering, Graduate School of Life Science and System Engineering, Kyushu Institute of Technology, 2-4 Hibikino, Wakamatsu-ku, Fukuoka, 808-0196 Japan; 4 Department of Bioprocess Technology, Faculty of Biotechnology and Biomolecular Sciences, Universiti Putra Malaysia (UPM), 43400 Serdang, Selangor Malaysia; 5 Laboratory of Processing and Product Development, Institute of Plantation Studies, Universiti Putra Malaysia (UPM), 43400 Serdang, Selangor Malaysia

**Keywords:** Environmental biotechnology, Microbial communities, Environmental microbiology

## Abstract

Microbial degradation of organic matters is crucial during the composting process. In this study, the enhancement of the composting of rice straw and chicken manure with biochar was evaluated by investigating the indigenous cellulolytic bacterial community structure during the composting process. Compared with control treatment, composting with biochar recorded higher temperature (74 °C), longer thermophilic phase (> 50 °C for 18 days) and reduced carbon (19%) with considerable micro- and macronutrients content. The bacterial community succession showed that composting with biochar was dominated by the cellulolytic *Thermobifida* and *Nocardiopsis* genera, which play an important role in lignocellulose degradation. Twenty-three cellulolytic bacterial strains were successfully isolated at different phases of the composting with biochar. The 16S rRNA gene sequencing similarity showed that they were related to *Bacillus* *licheniformis*, *Bacillus* *subtilis,* *Bacillus* *aerius*, and *Bacillus* *haynesii,* which were known as cellulolytic bacteria and generally involved in lignocellulose degradation. Of these isolated bacteria, *Bacillus* *licheniformis*, a facultative anaerobe, was the major bacterial strain isolated and demonstrated higher cellulase activities. The increase in temperature and reduction of carbon during the composting with biochar in this study can thus be attributed to the existence of these cellulolytic bacteria identified.

## Introduction

Solid waste management is crucial in reducing and eliminating the adverse effect of waste materials on human health and the environment. According to the world bank statistic, the global solid waste generated was 2.01 billion tons in 2016 and is expected to increase by 3.4 billion annually by 2050^1,2^. Effective treatment of these wastes will reduce their hazardous impacts and promotes sustainable development goals. Several technologies such as anaerobic digestion and composting have been developed for treating various solid waste generated from industrial, agriculture and livestock activities. Until now, composting has remained the most acceptable method used to treat animal manure because of its ability to remove pathogens, diminish odor generation, reduce the volume and moisture and improve storage and transportation options.

Composting is a simple biological process in which the organic materials are degraded naturally into stable humus-like substances facilitated by the indigenous microorganisms under aerobic conditions. The process is greatly assisted by microorganisms such as fungi, bacteria and actinomycetes which act as composting agents by decomposing the organic content of the substrate^3^. During the composting process, organic material such as lignocellulose is mostly decomposed by cellulases, which are primarily generated by cellulolytic bacteria. These bacteria have previously been detected at all phases of the composting process and play an important in degrading the lignocellulosic materials^4^. A well-carried out composting requires a mixture of ingredients that allow microbes to consume carbon and nitrogen. Therefore, the carbon to nitrogen (C/N) ratio is a crucial factor for the successful operation of the composting process. Fresh manure contains high nitrogen and moisture content which makes it hard to decompose properly. To adjust the C/N ratio and moisture content, manure was typically mixed with plant biomass such as rice straw, wood residues, and leaves. However, lignocellulose, the most abundant component of plant biomass consists of recalcitrant polymers such as cellulose, hemicellulose and lignin. One of the main challenges of the composting process is the difficulty of degrading the lignocellulosic material which will eventually affect the productivity and the quality of the compost product.

Recently, the addition of biochar into the composting system has received increasing interest due to its several advantages such as eliminating odor, reducing greenhouse gases and ammonia emissions, enhancing the degradation, humification and mineralization of organic matter^5,6^. The improvement of the composting process was due to the porous structure of biochar which facilitated the aeration of the compost mixture, stimulating microbial growth and activity^7^. Culture-independent techniques have been used to study the microbial diversity of composting with biochar as they provide more information about microbial composition. The latest studies were based on phenotypic characteristics and prediction of functional metabolism, which provide higher resolution in analyzing the microbial composition of the composting process. Although the culture-based method recovers a small portion of bacteria diversity, this technique is still required for describing the taxonomic and metabolic characteristics of individual strains. Previous studies demonstrated that the biochar addition could enhance the cellulolytic activity during the composting of poultry manure, chicken manure, and sewage sludge, respectively^8–10^. However, the studies were mainly based on identifying the relationship between the enzymatic activities and 16S rRNA profiling which provide inadequate information about the physical and functional characteristics of the isolated bacteria. Thus, the culture-dependent method remained an important tool and the best way to fully characterize the metabolic and physiological properties of the microbes. Despite the fact that Ravindran et al.^11^ and Chung et al.^12^ isolated cellulolytic bacteria during the composting of swine manure and chicken manure with biochar, no correlation analysis was performed in their work to explain the relationship between the bacteria and physicochemical parameters. Understanding the relationship between bacterial community and physicochemical characteristics of the compost would allow researchers to enhance the productivity of the composting process as well as the quality of its end-product. Therefore, in this study, the high-throughput 16S rRNA gene sequencing and isolation of cellulolytic bacteria at different stages of the composting process were carried out. In addition, the relationship of these bacteria with physicochemical properties was evaluated to explain their relevance in improving the composting process with biochar addition.

## Materials and methods

### Composting procedure

Chicken manure and rice straw were used as the main components for the composting process. The composting operation and the sampling process were carried out according to the method described by Zainudin et al.^13^. Briefly, the composting process was performed by mixing 150 kg (w/w) of chicken manure and 50 kg (w/w) of rice straw, equivalent to a 2:1 ratio, in a round high-density polyethylene bin with a diameter of 162 cm and height of 132 cm. The height of composting pile was around 100 cm. Composting was performed beneath the shed to prevent the effects of weather variations such as hot and rainy conditions. The rice straw used in this study was collected from the paddy field in Sekinchan, a district located on the west coast of peninsular Malaysia. The rice growers have given their consent for the acquisition of rice straw. The chicken manure was collected from the poultry farm at the Animal Research Center of the Institute of Tropical Agriculture and Food Security, UPM. Twenty percent (w/w) of oil palm empty fruit bunch (EFB) biochar was then mixed with the chicken manure and rice straw. The biochar used in this study was previously produced by Idris et al.^14,15^ at a temperature of 600 °C in a pool-type biochar reactor. The biochar characteristics are shown in Table 1. Another set of treatments without the addition of biochar was made as a control. The composting was done twice for each of the treatments due to the limited number of composting bins. The water content was set at 60% with no further adjustment of moisture until the composting process finish. The turning process was carried out every seven days to facilitate the aeration and homogenization of the composting material. The temperature was recorded by inserting the temperature meter probe (DMS-30LCD-1-5, DATEL, USA) at the core of the composting pile. The sample was collected during each of the turning processes. One representative composite sample was taken from a randomized location mainly from the middle and bottom part of the pile. The pH and moisture content of the compost was determined according to the method described by Wei et al.^16^ and Zainudin et al.^13^, respectively. Briefly, the pH was measured by mixing one gram of sample with 10 ml of distilled and the aqueous solution was then measured using a pH meter. The moisture content was measured by drying 10 g of the sample at 105 °C in a moisture analyzer (MX/MF-50 Moisture Analyzer, USA). The macronutrients: carbon (C) and nitrogen (N) were determined using LECO TruMac CNS elemental analyzer (MI, USA) while micronutrients: Phosphorus (P), Potassium (K), Calcium (C), Magnesium (Mg), Iron (Fe), Zinc (Zn), Copper (Cu) and Manganese (Mn) were detected using Inductively Coupled Plasma (ICP-OES, Perkin Elmer, USA). The analysis was performed based on a dry basis and each sample was measured in three replicates. All experimental methods were performed in accordance with relevant guidelines and regulations.

**Table 1:** Characteristics of EFB Biochar.

Parameters	Value
Carbon content^a^	55.7%
Hydrogen content^a^	2.7%
Oxygen content^a^	41.5%
Nitrogen content^a^	1.07%
Moisture^a^	3.6%
Ash^a^	28.0%
Volatile matter^a^	8.0%
Potassium^b^	34.8 g/kg
Phosphorus^b^	1.46 g/kg
Calcium^b^	3.26 g/kg
Magnesium^b^	1.24 g/kg

^a^Data from Idris et al.^15^.

^b^Data from Idris et al.^14^.

### High-throughput 16S rRNA gene analysis

DNA was extracted and purified from 1 g of the compost samples which was collected at different composting phases (thermophilic phase: day 7 and 14; mesophilic phase: day 21 and 28; cooling and maturing phases: day 35, 47, and 60) using the NucleoSpin^®^ soil DNA extraction kit (Machery-Nagel, GmbH & Co., Germany) according to the manufacturer instruction. The DNA extracted from composting replicates was pooled together during the preparation of sequencing analysis. The 16S metagenomic sequencing library was prepared by amplifying the 16S rRNA gene of the extracted DNA at 25 cycles using the KAPA HiFi Hot Start Ready Mix (Kapa Biosystems, Wilmington, MA, USA). The forward primer 341F (5′-CCTACGGGNGGCWGCAG-3′) and reverse primer 785R (5′-GACTACHVGGGTATCTAATCC-3′) was used to amplify the V3-V4 region of the 16S rRNA genes. In summary, the PCR amplicons were labeled with a dual adapter index containing a unique barcode sequence (Nextera XT Index kit). AMPure XP beads were used to purify PCR products with the index, and all samples were normalized to ensure an equal library was present in the pooled samples before library denaturing. As an internal control for MiSeq sequencing, a 30% spike-in of PhiX was applied. Finally, denatured pooled samples were placed into an Illumina 600 cycles V3 MiSeq reagent cartridge and run on the MiSeq instrument. Forward, Index 1, Index 2, and reverse reads were sequenced for 301, 8, 8, and 301 cycles, respectively. The PCR products were then analyzed using the Illumina MiSeq system.

### Isolation of mesophilic and thermophilic cellulolytic bacteria

The isolation of cellulolytic bacteria was done according to the method described by Zainudin et al.^17^. One gram of sample was added into the 50 ml tube containing 10 ml of Luria Bertani (LB) broth. The samples were serially diluted and were cultured onto LB and Tryptic Soy (TS) medium agar plate. The plates were then incubated at mesophilic (37 °C) and thermophilic (50 °C) temperatures for 12 to 24 h. The colonies grown on the agar plate were selected and streaked onto TS and LB medium agar plates containing 0.2% carboxymethyl-cellulose (CMC). The bacteria harboring the cellulolytic activity were identified as the strains that produce a clear zone around the colony. The isolated bacteria colony was repetitively sub-cultured to obtain a single pure culture.

### Cellulolytic activity assay

The enzymes activities of isolates were evaluated by point-inoculating the culture onto the surface of the TS and LB agar plate containing 0.2% CMC and incubated overnight at 37 °C and 50 °C, respectively. The cellulolytic activity was expressed as the substrate hydrolysis index. The index was calculated as the ratio between the diameter of the hydrolysis zone around the colony and the diameter of the colony in millimeter using the formula as follow:

$${\text{Cellulolytic index }} = \frac{{\left[ {{\text{Diameter of clear zone }}\left( {{\text{mm}}} \right) \, {-}{\text{ Diameter of bacteria colony }}\left( {{\text{mm}}} \right)} \right]}}{{{\text{Diameter of bacteria colony }}\left( {{\text{mm}}} \right)}} \,$$

### Identification of isolated strains

The identification of the isolates was done based on 16S rRNA gene sequencing. The Polymerase Chain Reaction (PCR) was performed based on the PCR colony method as described by Zainudin et al.^17^. A pair of universal primers 27F (5’-AGAGTTTGATCCTGGCTCAG-3’) and 1492R (5’-GGTTACCTTGTTACGACT-3’) targeting the 16S rRNA gene was used as forward and reverse primers. The amplification of the 16S rRNA gene was conducted in 50 μl of PCR Master Mix kit (QIAGEN) mixture containing 25 μl dNTP’s, 1.5mM MgCl_2_ buffer, DNA polymerase, 0.5 μl of each forward and reverse primers. The RNase-free water was added until the final volume reaches 50 μl. Thermocycling was set up according to the conditions provided by the kit protocol as follows: 3 min of initial denaturation at 94 °C, 0.5 min of denaturation at 94 °C, 0.5 min of primer annealing at 60 °C, 1 min of primer extension at 72 °C and final elongation at 72 °C for 10 min. The PCR products were analyzed by 1.5% agarose gel electrophoresis and visualized using Gel red staining on a UV transilluminator. The PCR products were purified using Nucleospin PCR clean-up (Machery-Nagel, GmbH & Co., Germany) according to the manufacturer’s instructions before sequencing. The PCR products were then sequenced and the 16S rRNA sequences data were compared with those of other know species in the genebank database (http://blast.ncbi.nlm.nih.gov/Blast.cgi).

### Bioinformatics and statistical data analysis

Bioinformatics analysis was done according to the method described by Zainudin et al.^13^. Briefly, the sequences were trimmed and filtered by using the Lotus pipeline. Chimera checking and operational taxonomic units (OTUs) cluster were analyzed by UPRASE. The taxonomy of clustered OTU was assigned by Ribosomal Database Project (RDP) classifier with the Greengenes database v13.8. The relationships between dominant bacteria genera and isolated bacteria and the physicochemical properties of compost were explained by Principal component analysis (PCA). The PCA was conducted by using the XLSTAT-Ecology (version 2017.10.20, Addinsoft, Inc., Brooklyn, NY, USA). All of the data were statistically analyzed using SPSS statistical software version 26 by IBM Corp. A T-test was conducted to explain the significant differences between samples in each treatment.

## Results and discussion

### Composting characteristics

**Figure 1:**
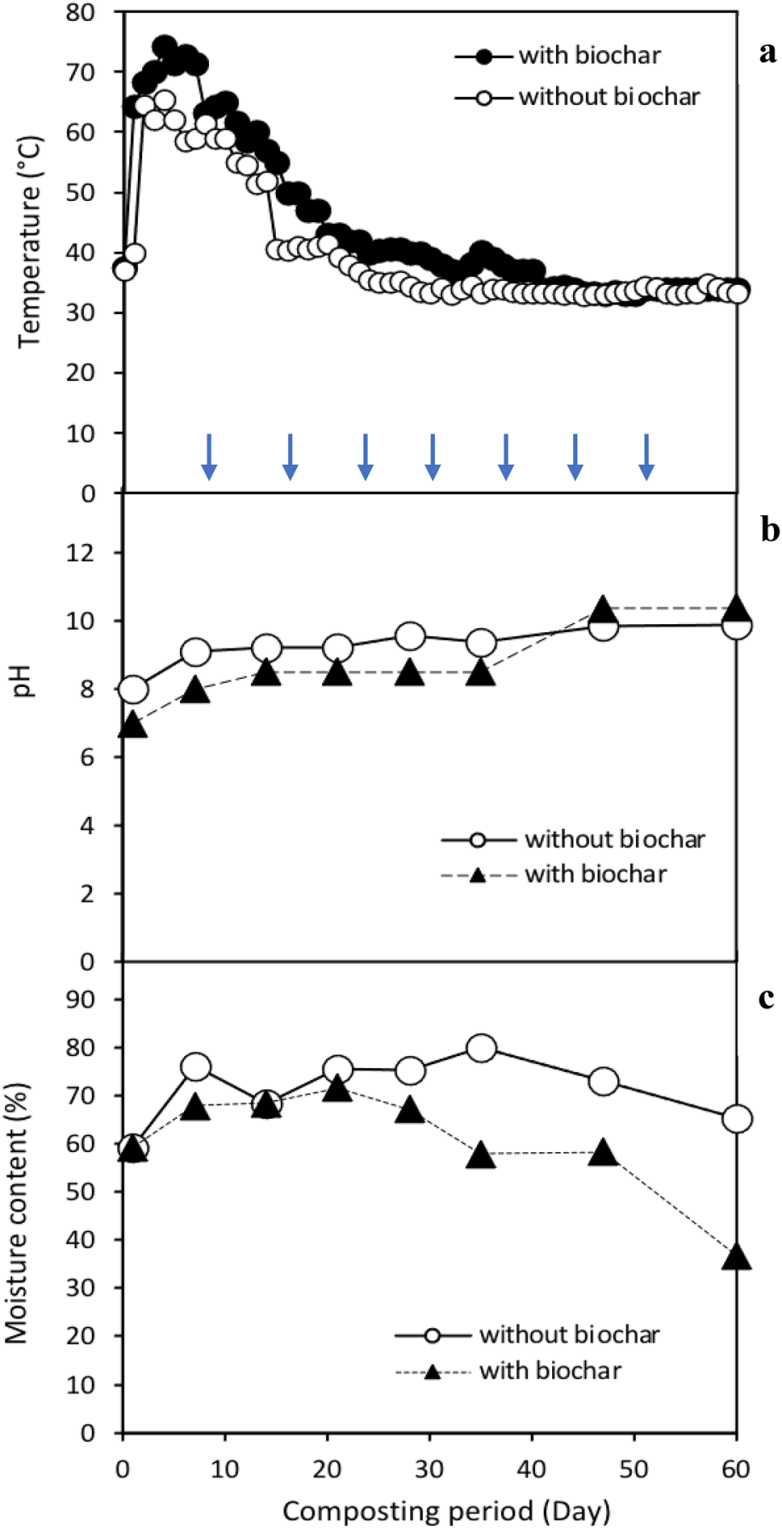
Profiles of temperature (**a**), pH (**b**) and moisture content (**c**) throughout the composting process with and without biochar addition. The blue arrows indicate the turning of composting pile. The data represent the average of replicated samples. The temperature was recorded by inserting the probe horizontally into the core of the compost pile (90 cm) at 3 different positions (upper, middle and lower). The pH was recorded from the combined sample taken from different sampling points (middle and bottom part of compost pile).

Table 2 shows the main characteristics of the composting process with and without the addition of biochar. The final composting process with biochar showed a significant (P<0.05) reduction of carbon, representing 19% of the initial C content, whereas in the composting without biochar, carbon reduction was only 8%. Both piles showed the escalation of the N content at the final stage of the composting process. However, the addition of biochar did not show any significant reduction (P>0.05) in the amount of N, supporting the hypothesis that biochar retains nitrogen in the composting mixture through the absorption of the nitrogenous compounds such as ammonia and ammonium onto the surface of biochar. Previous research has shown that composting with biochar reduces NH_3_ emissions while increasing NO_3_^–^ concentrations when compared to composting without biochar. According to Zainudin et al.^13^, the biochar addition to the composting pile improves the nitrification process, in which NH_4_^+^ is transformed into NO_3_^–^ by nitrifying bacteria. Furthermore, the oxidation of aromatic and carbonyl groups led to the creation of a positive and negative charge on the surface of biochar, promoting NH_4_^+^ and NO_3_^–^ adsorption^18^. The overall decrease in C content was higher in the biochar treatment pile than in the composting without biochar. In this study, composting was conducted by combining the chicken manure and rice straw at the ratio of 20:1 ratio, or C/N:15.6. According to Zhu^19^ and Zhou^20^, aerobic composting of swine manure with rice straw, edible fungal residue and rice bran at a low C/N ratio improves the maturation rate and increases organic matter degradation as compared to high C/N ratio composting. This finding is also in accordance with a previous study, which found that the dissolved organic carbon (DOC) was lower in biochar amended compost than without biochar due to the increased microbial activity^18^. It has been suggested in previous studies that adding biochar reduces the bulk density, which subsequently improves the aeration of the composting pile. The improved aeration promotes microbial proliferation and activity,thus, enhancing nutrient mineralization throughout the composting process. Thus, our finding supports the evidence of previous studies which reported that biochar improves organic matter degradation, hence, reducing the carbon content. Aside from the higher C reduction, the macro- and micronutrients such as phosphorus, kalium, calcium, magnesium, and zinc in composting with biochar were also higher than that of control composting. This could be due to the higher inorganic nutrient contents in the EFB biochar^14,15^. In addition, the organic coating forms on the outer and inner pore surfaces of biochar particles promotes the nutrient retention of the co-composted biochar^21^. The temperature of the composting process lasted for 14 days in control composting and 18 days in composting with biochar (Fig. 1a). The extended thermophilic phase is common for this organic material because rice straw contains recalcitrant compounds that are difficult for microorganisms to degrade, thus lengthening the composting process. However, the maximum temperature of the pile was higher in composting with biochar (74 °C) than in composting without biochar (62 °C), indicating that the addition of biochar enhanced the microbial activities, especially for the lignocellulose degradation. A study conducted by Huang et al.^22^ indicated that composting of pig manure and sawdust with an initial C/N of 15 resulted in a gradual rise in temperature, lower maximum temperature, and shorter thermophilic phase. However, it is interesting to demonstrate in our study that higher maximum temperature, rapid temperature increase, and longer thermophilic period can be achieved in composting with biochar at a low C/N ratio, implying the function of biochar in promoting the composting process as a result of higher organic matter degradation by microbes.

**Table 2:** Characteristics of initial mix and final product of rice straw and chicken manure composting with and without biochar addition.

	g/kg									
	C*	N	P*	K*	Ca*	Mg*	Fe*	Zn*	Cu	Mn
**Composting without biochar**										
Initial composting (day 7)	30.5 ± 0.34	2.28 ± 0.07	0.34 ± 0.03	0.86 ± 0.00	1.41 ± 0.01	0.41 ± 0.01	1.48 ± 0.22	0.81 ± 0.01	0.10 ± 0.04	0.23 ± 0.05
Final composting (day 60)	28.5 ± 0.46	2.55 ± 0.14	0.81 ± 0.00	3.29 ± 0.22	2.98 ± 0.10	0.78 ± 0.01	2.79 ± 0.14	0.63 ± 0.00	0.23 ± 0.01	0.93 ± 0.04
Percentage difference	7.6 ± 0.5	15.2 ± 5.1	139.1 ± 0.5	282.0 ± 19.9	112.1 ± 25.5	90.3 ± 3.1	87.4 ± 16.7	22.4 ± 1.2	108.5 ± 14.9	310.8 ± 22.0
(% reduction/increment)										
**Composting with biochar**										
Initial composting (day 7)	31.2 ± 0.19	1.99 ± 0.00	0.46 ± 0.01	4.00 ± 0.08	1.78 ± 0.07	0.88 ± 0.01	4.48 ± 0.40	0.44 ± 0.01	0.21 ± 0.01	0.60 ± 0.02
Final composting (day 60)	25.2 ± 0.23	2.34 ± 0.01	0.72 ± 0.04	4.10 ± 0.10	3.15 ± 0.27	1.06 ± 0.03	6.80 ± 0.52	0.88 ± 0.05	0.40 ± 0.02	0.86 ± 0.04
Percentage difference	19.2 ± 0.2	17.6 ± 0.7	56.7 ± 11.0	1.9 ± 0.5	77.1 ± 8.5	20.4 ± 1.3	52.1 ± 2.1	103.2 ± 16.0	83.9 ± 1.2	44.6 ± 2.2
(% reduction/increment)										

The symbol (*) indicates a significant difference at P** ≤ **0.05 between the samples.

The pH of the pile increased, whereas the moisture decreased as the composting progressed (Fig.1b,c). The results corresponded with a previous study which reported that the biochar addition increased the pH of the composting pile, which was due to the alkalinity properties of the biochar^23^. The physicochemical properties suggest that the enhancement of composting process could be due to the addition of biochar which improves the microbial activities for organic matter degradation. Therefore, in this study, we attempt to identify the bacterial community structure using high-throughput 16S rRNA gene sequencing and isolation of cellulolytic bacteria to evaluate further their relevance in improving the composting process with biochar.

### Bacterial community structure

**Figure 2:**
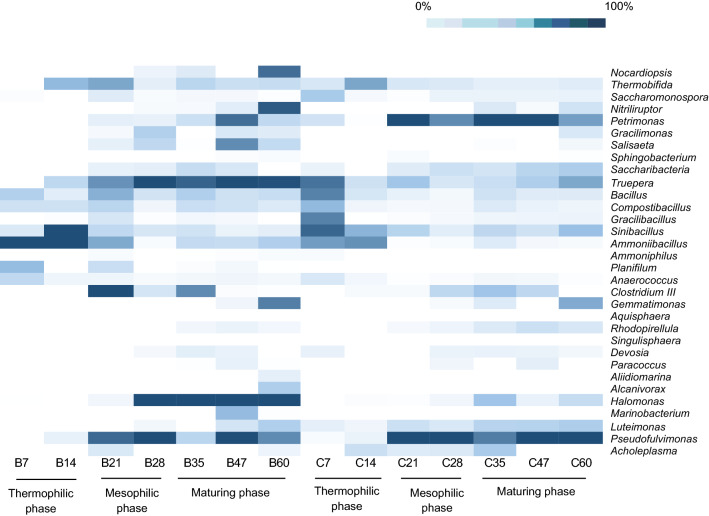
Heatmap analysis of the bacterial genera with the relative abundance of ≥ 0.1%. The (B) label denotes composting with biochar, while the (C) label denotes composting without biochar.

The high-throughput 16S rRNA gene sequencing was performed to clarify detailed information about the bacterial population during both of the composting processes. The heatmap analysis showed that the composting with biochar was generally dominated by the *Thermobifida*, *Nocardiopsis*, *Compostibacillus*, *Ammonibacillus,* *Sinibacillus*, *Bacillu*s, *Truepera*, *Halomonas*, *Pseudofulvimonas,* respectively (Fig. 2). These bacteria were often discovered during agricultural waste composting^13,24–26^. During the thermophilic phase, the most prevalent taxa were *Sinibacillus*, *Bacillus*, *Compostibacillus* *and* *Thermobifida.* However, the abundance of *Sinibacillus*, *Bacillus,* *and* *Thermobifida* decreased as the composting progressed. In contrast, the abundance of *Nocardiopsis* increases when the composting enters the mesophilic and mature phases. *Thermobifida* and *Nocardiopsis* were highly abundant in the composting with biochar as compared to composting without biochar.

In addition, the abundance of *Bacillus*, *Sinibacillus* and *Compostibacillus* during the thermophilic phase of the composting with biochar was higher than that of composting without biochar. These bacteria were able to live at the high temperature of the composting pile due to their ability to generate endospores. A previous study showed that members of *Bacillus* *and* *Sinibacillus* were mainly responsible for the degradation of high-molecular-weight of organic matter throughout the composting process^24^. Moreover, *Compostibacillus* which was previously isolated from sludge composting has been found as a facultative anaerobe and is capable of growing at temperatures as high as 60 °C^27^. Facultative anaerobes can grow and metabolize in aerobic and anaerobic environments although they favor oxygen-rich conditions^28^. Therefore, the increased abundance of *Compostibacillus* supports the finding of previous studies that the addition of biochar enhanced the aeration of the composting pile, thus, encouraging the development of bacteria including facultative anaerobe. *Truepera*, *Halomonas* and *Pseudofulvimonas* genera which are known as nitrifying and denitrifying bacteria^13^ showed an increasing trend as the composting entered the mesophilic and maturing stages. These bacteria were found to be highly dominant in the composting with biochar.

### Cellulolytic bacteria

Composting with biochar resulted in a greater reduction of carbon content, which might have been assisted by the presence of cellulolytic bacteria. As a result, in this study, we aim to isolate as many cellulolytic bacteria as possible from the composting with biochar. The isolation of cellulolytic bacteria was also done from the composting without biochar for comparison. The results showed that the number of isolated cellulolytic bacteria was greater in composting with biochar than in composting without biochar (Table 3). Twenty-eight of the isolated strains were found to be more than 99% identical to known cellulolytic bacteria. These strains were closely related to *Bacillus* *licheniformis*, *Bacillus* *subtilis,* *Bacillus* *aerius*, *Bacillus* *haynesii,* all of which were known to exhibit cellulolytic activity and have been involved in the lignocellulose degradation process^29–31^. Of these cellulolytic strains, *B.* *licheniformis* was the primary species isolated from the composting with biochar. The results corresponded with 16S rRNA sequencing data, which indicated that *Bacillus* was among the dominant bacteria detected throughout the composting process. *B.* *subtilis* had previously been isolated from soil and compost and was known to demonstrate cellulolytic activities^32,33^.

**Table 3:** Cellulolytic bacteria isolated at different stages of composting with and without the addition of biochar.

Type of composting/period (day)	Isolation temperature	Isolate ID	Cellulolytic index	NCBI BlastN similarity (%)
**Composting with biochar**				
7	50 °C	BTC4_7D	1.1	*Bacillus* *licheniformis* DSM 13 (100)
		BTC6_7D	0.5	*Bacillus* *licheniformis* DSM 13 (95)
21		BTC18_21D	1.5	*Bacillus* *licheniformis* ATCC 14580 (100)
		BTC10_21D	0.5	*Bacillus* *licheniformis* ATCC 14580 (99)
28		BTC11_28D	0.9	*Bacillus* *licheniformis* ATCC 14580 (99)
35		BTC3_35D	2.2	*Bacillus* *licheniformis* ATCC 14580 (100)
		BTC22_35D	0.1	*Bacillus* *licheniformis* ATCC 14580 (99)
47		BTC5_47D	0.4	*Bacillus* *aerius* *strain* 24K (100)
		BTC1_47D	0.8	*Bacillus* *haynesii* NRRL B-41327 (100)
		BTC6_47D	0.6	*Bacillus* *licheniformis* ATCC 14580 (99)
		BTC10_47D	0.7	*Bacillus* *licheniformis* ATCC 14580 (99)
		BTC11_47D	0.7	*Bacillus* *licheniformis* DSM 13 (99)
		BTC12_47D	0.5	*Bacillus* *licheniformis* DSM 13 (99)
		BTC17_47D	0.3	*Bacillus* *licheniformis* DSM 13 (99)
60		BTC18_F	0.5	*Bacillus* *licheniformis* DSM 13 (99)
		BTC19_F	0.6	*Bacillus* *licheniformis* ATCC 14580 (99)
		BTC21_F	0.4	*Bacillus* *subtilis* IAM 12118 (100)
		BTC7_F	0.5	*Bacillus* *licheniformis* DSM 13 (100)
		BTC8_F	1.0	*Bacillus* *licheniformis* DSM 13 (100)
7	37 °C	BMC8_7D	2.7	*Bacillus* *aerius* *strain* 24K (99)
35		BMC24_35D	1.0	*Bacillus* *licheniformis* DSM 13 (100)
35		BMC76_35D	0.5	*Bacillus* *subtilis* IAM 12118 (99)
47		BMC2_47D	1.3	*Bacillus* *licheniformis* DSM 13 (100)
**Composting without biochar**				
21	50 °C	TCC48_21D	0.8	*Bacillus* *licheniformis* DSM 13 (99)
35		TCC4_35D	0.7	*Bacillus* *haynesii* NRRL B-41327 (99)
		TCC10_35D	2.2	*Bacillus* *haynesii* NRRL B-41327 (99)
47		TCC11_47D	0.7	*Bacillus* *licheniformis* DSM 13 (100)
21		MCC11_21D	0.4	*Bacillus* *licheniformis* DSM 13 (100)
60		MCC12_60D	0.8	*Bacillus* *licheniformis* DSM 13 (99)

This bacterium has been widely used in various kinds of applications including cellulase production for saccharification and as an inoculum to enhance the composting process^34,35^. *B.* *aerius* is a thermophilic cellulolytic bacterium that was previously isolated from lignocellulosic waste and hot-spring sediment^30,36^. This bacterium plays a major role in the hydrolysis of lignocellulose and has been found to produce highly thermostable cellulases^37^.

### Relationships between physicochemical properties and bacterial community

**Figure 3:**
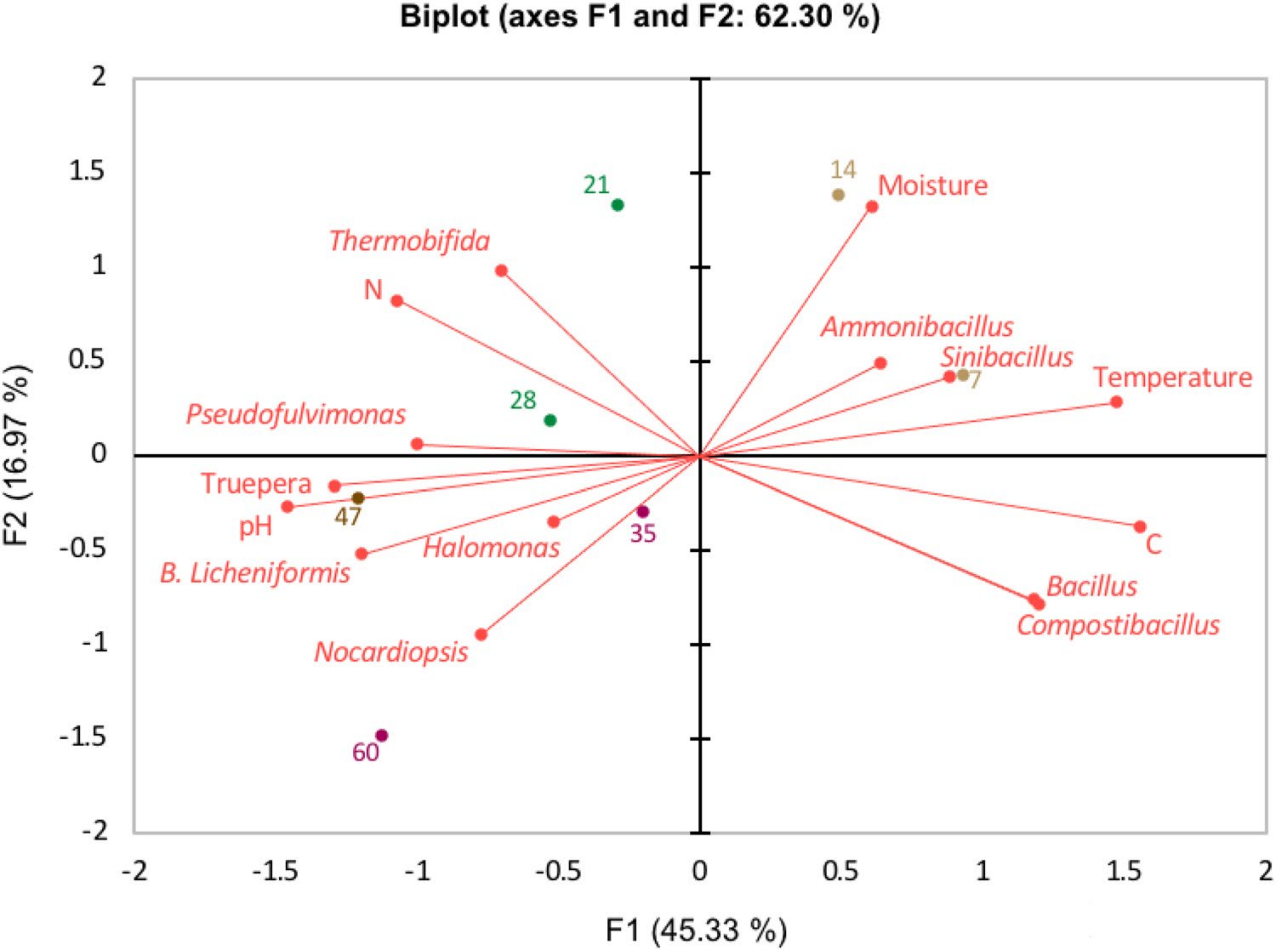
Principal component analysis (PCA) indicates the correlation and distribution of the samples between dominant phyla and physicochemical parameters during the composting with biochar. Interpretation of the data analysis was explained by Biplot. Original variables (dominant phyla and physicochemical characteristics) drawn as vectors (red line) was used to summarize the correlation between the variable. Clustering of the sample site due to the bacteria community composition and composting stages are highlighted by green, brown, and dark red dots. F1 and F2 axes represent 62.3% of the explained variance.

Since composting is a microbially-mediated process, understanding the relationship between microbe and physicochemical properties of the compost will substantially improve the efficiency of the composting process and the quality of its end-product. Therefore, a principal component analysis (PCA) was performed to explain the relationship between the dominant bacteria community, particularly cellulolytic bacteria, and physicochemical characteristics during the composting process with biochar. The results showed that *Thermobifida* *and* *Nocardiopsis* exhibited positive correlations with N content and pH, respectively (Fig. 3). In contrast, they showed negative correlations with C content, indicating their important roles in organic matters degradation, particularly the lignocellulosic materials, thus, reducing the C content as the composting progressed. *Thermobifida* and *Nocardiopsis* genera were known as cellulolytic bacteria and had been involved in the lignocellulose degradation^17,25^. They were important cellulolytic actinobacteria capable of generating a variety of hydrolytic enzymes for lignocellulose degradation, including exoglucanase, endoglucanase, β-glucosidase and xylanase. The majority of the enzymes produced by these bacteria were very stable at a broad range of pH and temperature^38,39^. These bacteria are aerobic, gram-positive and spore-forming bacteria and these traits are critical for their survival in harsh environments.

Similar to *Thermobifida* and *Nocardiopsis*, the cellulolytic *B.licheniformis*, also showed an inverse correlation with the C content, indicating its function in the degradation of the lignocellulose matrix. On the other hand, *B.licheniformis* positively correlated with pH but negatively correlated with moisture content and temperature. *B.* *licheniformis* is a facultatively anaerobic, gram-positive bacterium that has been isolated in a wide range of temperature environments^40^. In this study, *B.* *licheniformis* was successfully isolated at mesophilic and thermophilic temperatures, indicating that this bacterium can grow and thrive in both conditions^41^. According to He et al.^42^, *B.* *licheniformis* would grow fast and require less doubling time in the presence of oxygen. As suggested earlier, the purpose of adding biochar into the composting mixture was to lower the bulk density of the components, hence, enhancing the oxygen penetration to the interior part of the pile. The high-water content of the composting without biochar (Fig. 1c) might saturate the system and reduce the oxygen penetration, thus, decreasing the *B.* *licheniformis* population. As a result, the reduced moisture content of the composting with biochar enhanced the compost environment for the *B.* *licheniformis* development. Previous studies reported that the micro-aerobic pretreatment of lignocellulose corn straw and microalgal biomass increased the activity of the hydrolytic enzymes such as cellulase and encouraged the development of facultative anaerobic bacteria^42,43^. Furthermore, *B.* *licheniformis* isolated from bovine rumen had the highest cellulose-degrading activity under micro-aerophilic conditions^44^. This corresponds with our earlier discussion that the increasing number of cellulolytic facultative anaerobe *B.* *licheniformis* could be attributed primarily to adequate oxygen supplementation for this bacterium to thrive as a result of the large surface area and porosity of biochar, which produces the micro-aerobic conditions inside of the composting pile. In addition, *Truepera*, *Halomonas*, *Pseudofulvimonas* also showed a negative correlation with C content, indicating that the increased abundance of these genera could be attributed to the consumption of readily utilizable organic fraction generated as a result of the organic matter degradation during the thermophilic stages, hence, reducing the carbon content towards the end of the composting process. Overall, the findings of this study suggest that the enhancement of the composting with biochar was due to the abundance of cellulolytic bacteria detected, which may have led to higher organic matter degradation especially the lignocellulosic material throughout the composting process.

## Conclusion

The treatment with biochar showed an increase in the temperature of composting pile and reduced the carbon content while retaining a considerable amount of micro- and macronutrients as compared to composting without biochar. High-throughput 16S rRNA gene analysis demonstrated that the thermophilic stage was dominated by cellulolytic bacteria related to the *Thermobifida* genus, while the *Nocardiopsis* genus dominated the mesophilic and maturing stages. Isolation of cellulolytic bacteria also indicated that the strains related to the facultative anaerobe *B.* *licheniformis,* *B.* *subtilis,* *B.* *aerius* and *B.* *haynesii* strains were identified at different stages of the composting process. The finding of this study showed that the cellulolytic bacterial community was significantly correlated with changes in physicochemical properties, notably the C content, suggesting that their existence might be the reason for the composting process’s improvement.
